# Role of bioaerosol in virus transmission and material‐based countermeasures

**DOI:** 10.1002/EXP.20210038

**Published:** 2022-05-23

**Authors:** John Joseph, Helna Mary Baby, Spencer Zhao, Xiang‐Ling Li, Krisco‐Cheuk Cheung, Kabir Swain, Eli Agus, Sruthi Ranganathan, Jingjing Gao, James N Luo, Nitin Joshi

**Affiliations:** ^1^ Center for Nanomedicine, Department of Anesthesiology Perioperative and Pain Medicine, Brigham and Women's Hospital Boston Massachusetts USA; ^2^ Harvard Medical School Boston Massachusetts USA; ^3^ Department of Surgery Brigham and Women's Hospital Boston Massachusetts USA

**Keywords:** bioaerosol, COVID‐19, nanomaterial, respiratory droplet, resuspension, secondary aerosolization

## Abstract

Respiratory pathogens transmit primarily through particles such as droplets and aerosols. Although often overlooked, the resuspension of settled droplets is also a key facilitator of disease transmission. In this review, we discuss the three main mechanisms of aerosol generation: direct generation such as coughing and sneezing, indirect generation such as medical procedures, and resuspension of settled droplets and aerosols. The size of particles and environmental factors influence their airborne lifetime and ability to cause infection. Specifically, humidity and temperature are key factors controlling the evaporation of suspended droplets, consequently affecting the duration in which particles remain airborne. We also suggest material‐based approaches for effective prevention of disease transmission. These approaches include electrostatically charged virucidal agents and surface coatings, which have been shown to be highly effective in deactivating and reducing resuspension of pathogen‐laden aerosols.

## Graphical abstract

Virus dissemination through droplets or aerosols is a predominant transmission mode of respiratory infections. This review summarizes direct and indirect mechanisms of aerosol generation with an emphasis on infection risks presented by the resuspension of settled droplets. Material‐based approaches, including nanotechnology, to contain bioaerosol‐mediated virus transmission are highlighted in this review.

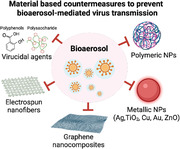

## 1 INTRODUCTION

**FIGURE 1:**
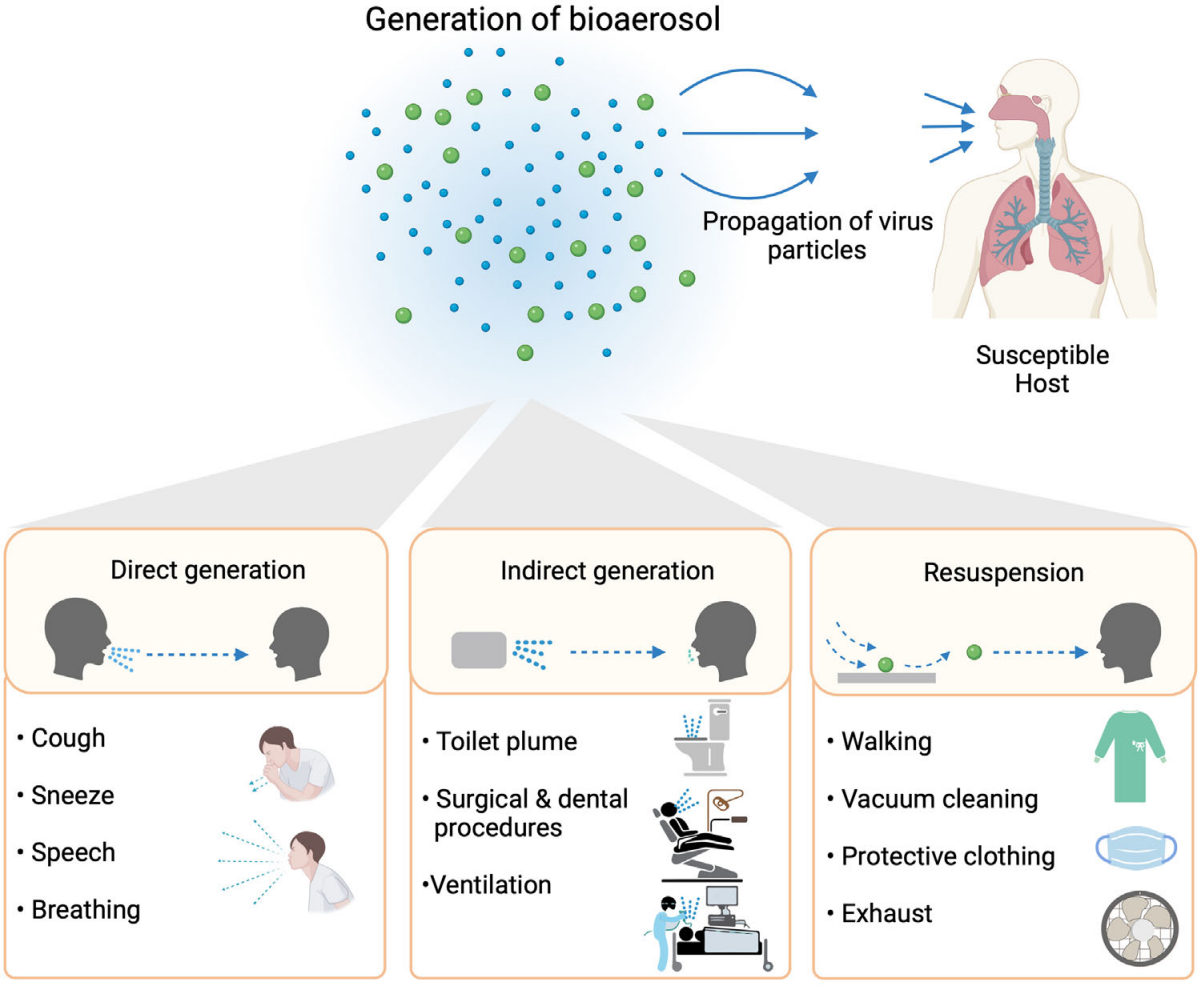
Modes of transmission. Bioaerosol can disseminate virus particles and induce the infection over long distances beyond 1 m. Bioaerosols are generated in three different ways: (1) Direct generation of bioaerosols from the infected individuals during coughing, sneezing, talking, or even normal breathing; (2) indirect generation during toilet flushing and medical procedures such as mechanical ventilation, intubation, and cardiopulmonary resuscitation; (3) Resuspension of settled droplets due to the mechanical forces produced during walking, vacuum cleaning, doffing of protective apparels, or high airspeed of exhaust fans

Respiratory infections are one of the most common diseases globally, owing to the ease of transmission through respiratory droplets and aerosols (generally referred to as bioaerosols). The ability of bioaerosols to travel long distances and linger in air for a long duration enables dissemination of disease, causing outbreaks such as the COVID‐19 pandemic. Viruses embedded in mucus and saliva are expelled while talking, breathing, coughing, and sneezing. A large number of evidence‐based studies during the COVID‐19 pandemic have demonstrated the transmission of SARS‐CoV‐2 via bioaerosols.^[^ ^1^, ^2^ ^]^ The size of virus‐laden bioaerosols is a determinant factor that governs settling time, travel distance, and transmissibility. The World Health Organization (WHO) and Centers for Disease Control and Prevention (CDC) classify particles with an aerodynamic diameter greater than 5 µm as droplets, while particles less than or equal to 5 µm are termed aerosols or droplet nuclei.^[^ ^3^ ^]^ As shown in Figure 1, pathogen‐containing bioaerosols can cause transmission directly or indirectly via primary bioaerosols or secondary bioaerosols resulting from resuspension of settled droplets. The trajectory and transmissibility of pathogen‐laden primary aerosols are complex and can be affected by multiple factors, including environmental conditions such as temperature and relative humidity. The viability and survival time of pathogens in bioaerosols are also influenced by environmental conditions and the mechanisms of bioaerosol generation. Although multiple studies have looked at primary bioaerosols and their role in disease transmission, less attention has been paid to the risk posed by secondary bioaerosols, which can contribute significantly to the spread of respiratory diseases. This is especially true when the pathogen is highly infectious and can survive for several hours on surfaces. Multiple studies have demonstrated the role of bioaerosol resuspension in spreading airborne pathogens. For instance, in the series of anthrax attacks in 2001, the letter containing *Bacillus anthracis* spores was promptly removed after it was opened.^[^ ^4^ ^]^ However, reports later showed that airborne anthrax was detected in the suite even after 25 days. This was explained by the resuspension of spores that had settled onto surfaces during the initial incident. A greater understanding of bioaerosol resuspension is therefore critical to assess the transmissibility of pathogens. It can provide critical insights into the prevalence and dominance of certain viruses, such as the Delta and Omicron variants of SARS‐CoV‐2, which have more positively charged spike proteins that can lead to longer survival time in aerosols.^[^ ^5^ ^]^ Notably, positive charge is speculated to increase the interaction of these variants with the negatively charged glycoproteins of mucin found in the droplets. Mucin can therefore form a thin, cross‐linked network that encapsulates and protects the virus in aerosol, leading to longer survival times and posing a greater risk for resuspension.^[^ ^6^ ^]^

In this review, we discuss different mechanisms of bioaerosol generation with an emphasis on sources of bioaerosol, critical factors that impact their trajectories and transmissibility, and the associated epidemiological risk. Finally, we discuss countermeasures to prevent viral infections via bioaerosol. In particular, we discuss virucidal materials which can be grafted onto different substrates, painted onto surfaces, or applied as a coating to personal protective equipment (PPE), such as medical masks. Given the risk posed by bioaerosol resuspension, measures and materials that can reduce the viability of deposited pathogens could be instrumental in preventing the further spread of respiratory infections. The scope of this review also underlines the significance of bioaerosol resuspension, which is often overlooked as a mode of transmission. We envision that this review will reignite material scientists to develop broad‐spectrum antiviral technologies to combat future outbreaks.

## 2 DIRECT GENERATION OF PRIMARY BIOAEROSOL

Pathogen‐laden bioaerosols are generated directly from infected individuals during expiratory activities such as breathing, talking, sneezing, and coughing. These activities generate respiratory droplets varying in both size and viral load. Prior research suggests regular nasal breathing and exhalation discharge submicron and larger droplets.^[^ ^7^ ^]^ Coughing and sneezing generate 3000 droplet nuclei and 40,000 droplets, respectively,^[^ ^8^ ^]^ but coughing expels the highest concentration of droplet nuclei smaller than 1 µm.^[^ ^9^ ^]^ The number of viral particles found in a droplet can range from 10^2^ to 10^11^ copies/mL.^[^ ^10^ ^]^ Theoretically, viral load in the droplets depends on the cube of droplet diameter; however, some studies suggest that smaller droplets may be virus‐enriched.^[^ ^11^ ^]^ The widely used assumption is that the pathogenic particle distribution in a droplet follows a Poisson distribution.^[^ ^10^ ^]^ Using a Monte‐Carlo simulation, Wang et al. estimated that a person with a viral load of 2.35 × 10^9^ copies/mL could generate 1.23 × 10^5^ copies of airborne virus by a single cough.^[^ ^12^ ^]^ The viral load required to trigger an infection depends upon the type of virus. Alford et al. conducted a study to determine the minimum infectious aerosol dose of influenza A2/Bethesda/10/63 in 23 volunteers aged between 21 and 40 years. Volunteers were subject to inhalation of 10 L of aerosolized virus suspensions by face mask. Subjects free of serum neutralizing antibodies were infected by a dose of approximately three tissue culture infectious dose 50 (TCID50),^[^ ^13^ ^]^ which enumerates 1.95 × 10^3^ viral copies based on subsequent studies estimating 300–650 copies of human influenza viruses in 1 TCID50.^[^ ^14^ ^]^ A separate survey by Memoli et al. sought to determine the dose of influenza A(H1N1)pdm09 needed to induce mild to moderate infection in 46 healthy volunteers following intranasal exposure. In this study, an amount of 10^7^ TCID50 induced influenza infection in 69% of participants.^[^ ^15^ ^]^ More recently, there has been an interest in establishing the relationship between viral load and infectivity in SARS‐CoV‐2. A mathematical model by Goyal et al. estimates infectious dose 50 (ID50) of SARS‐CoV‐2 to be 10^7^ viral RNA copies.^[^ ^16^ ^]^

**FIGURE 2:**
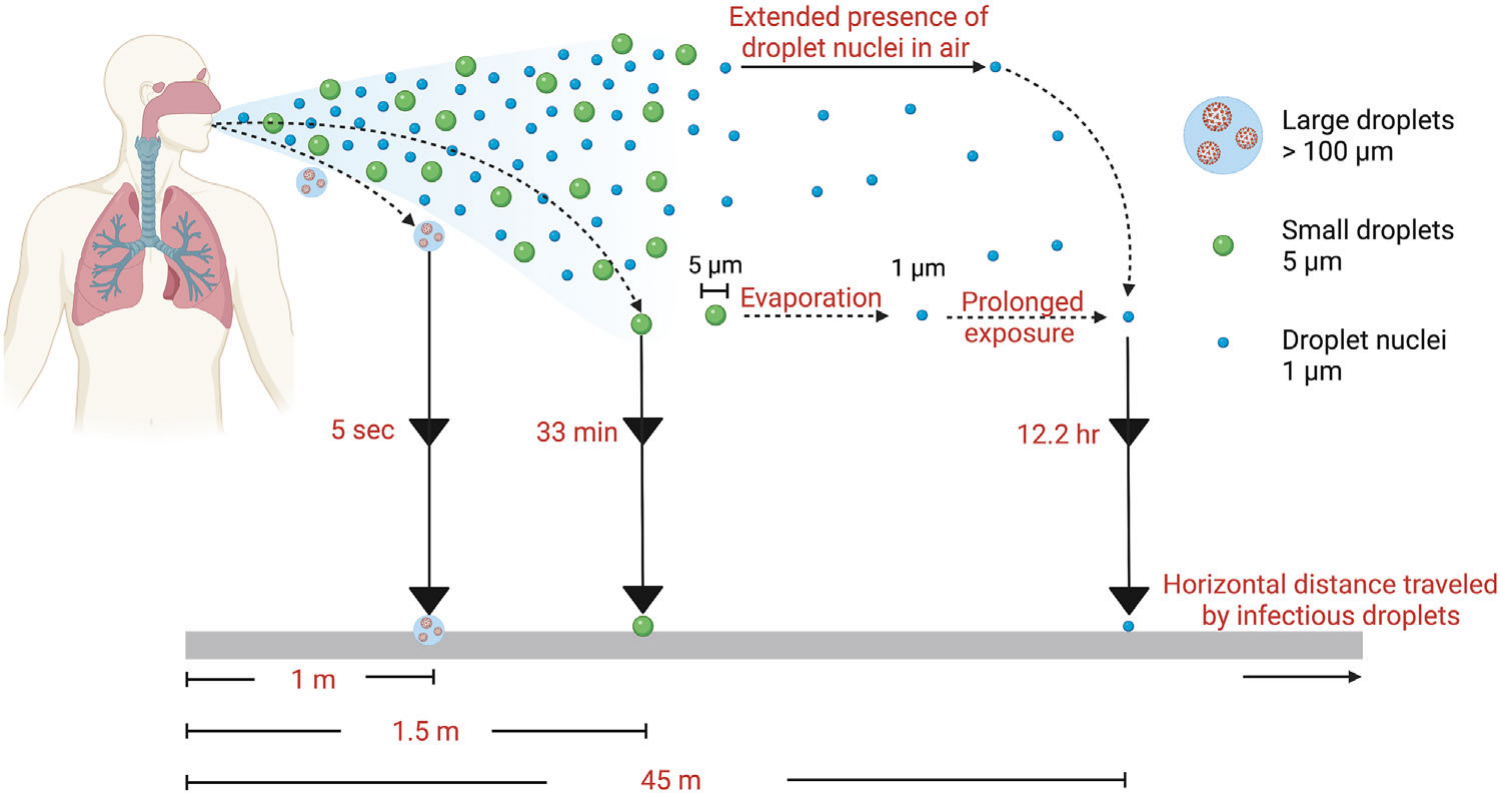
Trajectory, settling time, and conversion of respiratory droplets to droplet nuclei. Respiratory events such as sneezing and coughing generate a population of virus‐laden droplets of different sizes, namely large droplets (>100 um), small droplets (<5 um), and droplet nuclei (<1 um). Larger droplets experience gravitational forces and settle down at 1 m within a short time of 5 s. Small droplets can remain in the air for 30 min and travel beyond 1 m. These droplets may also undergo evaporation and decrease in size to form droplet nuclei or bioaerosols. Droplet nuclei drift in the atmosphere for a prolonged duration (12 h) up to 45 m and pose a high risk of infections at long range

Besides viral load, horizontal distance traveled and time of flight are important factors in determining the transmissibility of aerosols. Studies have revealed that droplets expelled from breathing and coughing can travel distances up to 3–6 feet with speeds less than 10 m/s, while droplets generated from sneezing can propagate 20 feet or more at 50 m/s.^[^ ^17^, ^18^ ^]^ In contrast, large droplets (greater than 100 µm) settle down rapidly, contaminating the zone around the host. The relationship between droplet size, duration of suspension in air, and trajectory is shown in Figure 2. The trajectory of a droplet is influenced by the equilibrium between gravitational and upward Stoke's drag force. Large droplets experience more gravitational pull than drag force, leading to short settling times in a few seconds.^[^ ^19^ ^]^ At a height of 1.5 m, larger droplets travel no farther than 1 m before settling down. The terminal velocity of respiratory droplets also affects the horizontal distance they can travel. In contrast to large droplets, the low terminal velocity of small droplets lesser than 5 µm allows them to be suspended in air for more extended periods in conjunction with air currents.^[^ ^20^ ^]^ Therefore, airflow can facilitate small droplets to reach high altitudes, such as ventilation systems, thereby increasing the incidence of airborne transmission. A recent report validated this by showing the existence of SARS‐CoV‐2 droplets from the ventilation systems in hospital rooms of infected patients.^[^ ^21^ ^]^

Evaporation also impacts the travel distance and transmission of virus‐laden respiratory droplets. A recent study demonstrated that droplets in the range of 12–21 µm, generated during a loud speech, evaporated to produce droplet nuclei or aerosols (1 µm) within 15 min in an indoor setting. The decrease in size of evaporated droplets is accompanied by a slow terminal velocity and they remain airborne for longer durations.^[^ ^22^, ^23^ ^]^ This is further exemplified in another study where the size reduction of a droplet from 50 µm to 10 µm experiences a decrease in terminal velocity from 6.8 cm/s to 0.35 cm/s.^[^ ^24^ ^]^ Apart from temperature, the evaporation rate is also determined by the differential vapor pressure between the droplet surface and the surrounding air.^[^ ^25^ ^]^ The magnitude of droplet size reduction during evaporation depends on the nonvolatile solute composition, typically electrolytes, deoxyribonucleic acid, sugar molecules, enzymes, and fragments of desiccated epithelial and white blood cells. The weight percentage of these non‐volatile substances in respiratory fluids ranges from 1% to 5%. Upon exposure to low relative humidity, aerosols composed of salt and organic material encounter liquid–liquid phase partition, resulting in a core–shell assembly.^[^ ^26^ ^]^ Hence, the amount of droplet evaporation and the concentration of the viral particles in the aerosol are influenced by humidity, as depicted in Table 1. Droplets evaporate much faster in low relative humidity (RH) and increase the infectivity by concentrating the pathogen load in the droplet nuclei. Smaller droplets evaporate rapidly, resulting in bioaerosols or droplet nuclei (1 µm) that can persist in the air for up to 12 h. In addition to transport kinetics, temperature and humidity can influence the viability of viruses in aerosols. However, the mechanisms by which humidity affects virus stability are not well elucidated. One hypothesis involves inactivation of enveloped viruses due to surface tension and conformational changes by hydrophobicity at the air–liquid interface. Higher RHs tend to increase final aerosol size and surface area due to hygroscopic effects, therefore leading to surface inactivation of enveloped viruses. Salt concentration in droplets has also been theorized to have a stronger dependence of humidity on virus viability. For example, there is minimal evaporation at RHs close to 100%. In this case, the salt concentration in the aerosol remains stable, and virus viability is uncompromised. At RHs greater than 50% but lesser than 100%, evaporation concentrates the salt levels within the droplets to be toxic, disrupting the viral membrane structure. Salt crystallization observed at RHs less than 50% does not affect virus viability.^[^ ^27^ ^]^

**TABLE 1:** Environmental factors influencing evaporation of respiratory droplets and virus transmission. Relative temperature, absolute humidity levels, air pressure, etc., have been shown to drastically affect the viral particle's ability to successfully infect an individual through the cascading effects it has on the amount of time an active viral particle is in the air

**Environmental factors**	**Description**
Lower relative temperature	The transmission of SARS‐CoV‐2 saw an increase when the temperature was lower^[^ ^120^ ^]^ Lower rates of SARS‐CoV inactivation^[^ ^121^ ^]^ Increased survival of SARS‐CoV virus on surfaces^[^ ^122^ ^]^ Reduced airborne time for viral loaded droplets^[^ ^123^ ^]^
Higher relative temperature	Transmissions were notably lower for SARS‐CoV‐2 when temperatures were higher^[^ ^120^, ^124^ ^]^ Increased inactivation of SARS‐CoV‐2 on surfaces^[^ ^123^ ^]^ Shorter droplet evaporation time prolonging duration before being deposited on a surface^[^ ^125^ ^]^
Lower absolute humidity	Environments with a relatively lower absolute humidity saw higher rates of transmission of SARS‐CoV‐2^[^ ^120^ ^]^ Prolonged airborne time for viral loaded droplets^[^ ^56^ ^]^
Higher absolute humidity	Areas with a relatively higher absolute humidity experienced slightly lower rates of transmission of SARS‐CoV‐2^[^ ^120^ ^]^ Reduced evaporation of droplets, shortening the time before being deposited on a surface^[^ ^123^ ^]^
UV (ultraviolet)/sunlight exposure	Virus and microorganism viability has been shown to be strongly affected by sunlight exposure, primarily due to radiation damage to genetic material^[^ ^38^ ^]^ One study showed a 15‐fold in decay constant and 15‐fold decrease in half‐life of Influenza A virus A/PR/8/34 under simulated sunlight^[^ ^38^ ^]^ Another study has demonstrated rapid inactivation of SARS‐CoV‐2 in aerosols under ground‐level sunlight^[^ ^3^ ^]^

Multiple reports have substantiated the influence of humidity and temperature on droplet lifetime and pathogen stability.^[^ ^28^, ^29^ ^]^ In one study, an enveloped virus ϕ6 was uniformly distributed in a desiccated droplet after rapid evaporation at low relative humidity. Viability experiments revealed that the high relative humidity favored adenovirus and rhinovirus stability in aerosols, whereas respiratory syncytial virus (RSV) exhibited dual‐mode stability at 20% and 40% to 60% RH.^[^ ^30^ ^]^ Dissemination of influenza virus under different RHs and ambient temperatures was studied in a guinea pig model.^[^ ^31^ ^]^ Higher transmission was observed at 5°C but reduced significantly at 30°C. High humidity (80% RH) was noticeably unsuitable for the transmission of influenza virus, although arid conditions of 20% and 35% RH were perceived as favorable. A few studies have also explored the critical role of absolute humidity in the survival of influenza virus and its transmission efficiency. Low absolute humidity levels during winter strengthen the virus survival and transmission.^[^ ^32^ ^]^ SARS‐CoV‐2 can survive for several days at moderately low temperatures (less than 20°C) and low absolute humidity.^[^ ^33^, ^34^, ^35^ ^]^ One mathematical study modeled the settling time, transport, and dehydration of SARS‐CoV‐2 containing droplets generated by cough under various RH and air velocities. Results showed that droplet size expanded due to the hygroscopic effect at high relative humidity (>80%) and enhanced droplets' deposition on the ground, whereas respiratory droplets evaporated at 40% RH to form droplet nuclei and persisted in the air.^[^ ^36^ ^]^ In another study, droplet lifetime was evaluated at different temperature and relative humidity conditions, and an exponential surge in droplet lifetime was observed at 30°C and 55.7% RH.^[^ ^37^ ^]^ Ultraviolet (UV) light exposure is also known to have a detrimental effect on virus viability, primarily mediated through damage to the viral genome,^[^ ^3^, ^38^ ^]^ as described in Table 1. Data from these studies highlight the importance of droplet nuclei formation in pathogen transmission and provide insight into the influence of factors such as temperature and humidity on virus viability.

## 3 INDIRECT GENERATION OF PRIMARY BIOAEROSOL

Primary bioaerosols can also be generated indirectly during medical procedures termed as aerosol‐generating procedures (AGPs).^[^ ^39^, ^40^, ^41^ ^]^ These procedures include bronchoscopy, non‐invasive ventilation (NIV), cardiopulmonary resuscitation, manual ventilation, and autopsy. Procedures such as NIV are categorized as AGP as they result in aerosolization of patient expirations due to high velocity gas flows. A study by Lavoie et al. investigated aerosol generation during bronchoscopy and showed a significant increase in aerosol production during the procedure compared to an empty room.^[^ ^42^ ^]^ However, risk of disease transmission from these AGPs is not well studied. A retrospective study on the nosocomial transmission risk factors of SARS showed that health workers caring for patients treated with NIV had two times increased risk of infection compared to medical staff who had no interaction with NIV‐treated patients. Despite this, the role of AGP is unclear; as the exact transmission route was not determined, the increased infection risk could instead be attributed to close contact with SARS patients. Given the infectious nature of bioaerosols generated from ill patients, there is an understandable concern among physicians and other healthcare workers performing AGPs. Further study into their transmission potential is warranted to ensure that appropriate personal protective equipment is used for these procedures.

Other indirect events of bioaerosol generation include toilet flushing. Toilet plumes have been studied previously in disseminating infectious diseases. The high speed of water flow from the tank creates a centripetal force that expels aerosols from the surface of the bowl, posing infection risks.^[^ ^43^ ^]^ It is also important to note that the aerosols produced by toilet flushing are within the respirable size range (<5 µm).^[^ ^44^ ^]^ In one study, a toilet bowl was seeded with *Serratia marcescens* and *Escherichia coli* to mimic bacterial shedding in stool. A significant number of airborne bacteria were detected after subsequent flushing.^[^ ^45^ ^]^ Likewise, norovirus persists in the air after toilet flushing and can be a secondary source of infection in closed environments.^[^ ^46^ ^]^ In another instance, virus particles were detected on floor surfaces from toilet stalls used by patients positive for SARS‐CoV‐2.^[^ ^21^ ^]^ These studies indicate that indirectly generated bioaerosols remain viable and can disperse in the air for up to 12 h, eventually settling on bathroom surfaces such as walls, floor, flush handle, sink, and cabinet. This underlines the importance of decontamination strategies in hospitals, houses, and other public areas to eradicate secondary sources of infection.

## 4 GENERATION OF SECONDARY BIOAEROSOL

**FIGURE 3:**
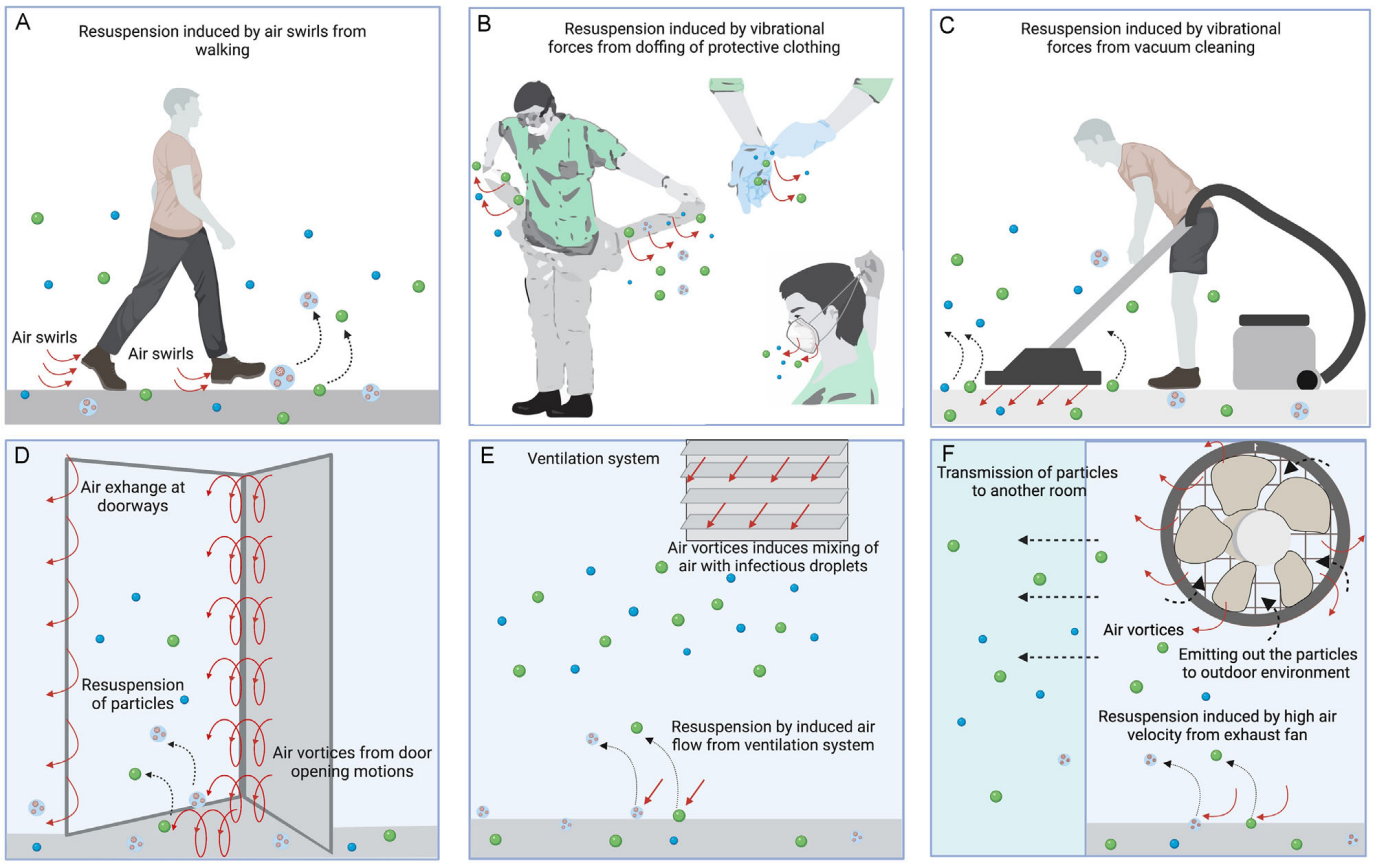
Events leading to the resuspension of bioaerosol. Resuspended virus‐laden particles can act as a secondary source of infections in indoor and outdoor settings. Particles are propelled off the substrate through mechanical vibrations or high air vortices experienced at the surface and suspended in air. (A) Air swirls generate around the legs while a person walks; (B) vibrational forces during doffing of personal protective equipment; (C) mechanical vibrations on floor surfaces from vacuum cleaning can lift the particles in the air. High air velocity and turbulence generated from the (D) door opening movements, (E) ventilation windows, and (F) exhaust fans re‐aerosolize the deposited droplets into the atmosphere. Air exchange leads to the propagation of virus particles into another room or outside environment, increasing the incidence of infection spread

Respiratory droplets settled on surfaces have a propensity to detach and resuspend in air through various mechanisms, as shown in Figure 3. Resuspended particles can then act as secondary sources of infection. Longer survival times on surfaces can also lead to a greater risk of secondary infection by means of aerosol resuspension. As discussed in Section 2, virus stability within the respiratory aerosols can differ significantly between viruses and depends on factors including humidity, temperature, UV exposure, and the chemical composition of aerosol itself. Based on environmental conditions and virus phenotype, the time required to achieve 99.99% virus deactivation can range from hours to periods as long as months.^[^ ^47^ ^]^ A study conducted to evaluate the stability of SARS‐CoV‐2 on different surfaces showed that SARS‐CoV‐2 was stable in aerosols for at least 3 h and remained viable on surfaces such as plastic and stainless steel for up to 3 days,^[^ ^48^ ^]^ thereby posing a high probability for the presence of infectious resuspended droplets.

Resuspension of a settled droplet occurs through a two‐phase process.^[^ ^49^ ^]^ The first phase involves the detachment of bioaerosol from the substrate, induced by hydrodynamic lift and vibrational forces leading to inceptive motions such as sliding, rolling, and liftoff.^[^ ^50^ ^]^ Several experiments reveal the dominance of rolling motion as the primary mechanism of particle detachment. The second phase involves resuspension of particles into bulk air streams. Not all dissociated particles become aerosolized; some detached particles transiently settle down due to the opposing gravitational force. In addition to gravitational force, van der Waals interactions, capillary forces, and electrostatic forces counteract resuspension forces to prevent detachment and secondary aerosolization of droplet particles.

### 4.1 Resuspension of bioaerosol by human activities

A critical contributing factor to bioaerosol resuspension is sporadic air perturbances from human activities in indoor and outdoor settings (Figure 3A).^[^ ^51^ ^]^ Stemming from the 2001 anthrax attacks, numerous studies have investigated the resuspension of *B. anthracis* spores. In one instance, wiped samples and surface dust were collected to quantify viable resuspended spores under simulated office conditions for 25 days after the initial outbreak. A remarkable increase was observed in the resuspension by events such as active foot trafficking, mail sorting, and moving of trash boxes. An airborne concentration of 100–11000 CFUs m^–3^ of B anthrax spores within the respirable range of 0.95–3.5 µm was identified.^[^ ^4^ ^]^ In another study, bioaerosol resuspension was assessed from personnel activity while maintaining a contaminated aerosol sampler.^[^ ^52^ ^]^ Air samples and swabs from the technician's clothing were investigated, and a significant concentration of 73 CFU/L_air_ was detected from the air and clothing. Another report revealed the resuspension of spores from mechanical sorting of contaminated envelopes, which led to the infection of employees.^[^ ^53^ ^]^ These studies demonstrate the substantial risk of resuspended *B. anthracis* spores by human activity. Resuspension of respiratory droplets also disseminated influenza virus by air turbulences produced during walking.^[^ ^54^ ^]^ In a previous report, the magnitude of air turbulence was measured in eddy diffusion coefficients. The eddy diffusion coefficients, determined experimentally, were applied to a simulated atmospheric transport model to estimate the concentration of resuspended influenza virus. Eddy diffusion coefficients were highest at 1 m from the floor. Therefore, around 40% of resuspended virus‐laden droplets were highly dispersed at the same height. Moreover, the study interpreted the possibility of different exposure levels of individuals to resuspended aerosols based on body height. People with a height of less than 1 m were more prone to exposure to resuspended viruses. In general, the influenza virus has superior stability and remains viable in outdoor or indoor environments for hours to days. Therefore, resuspension of settled influenza virus droplets could be a potential secondary disease transmission mode. In addition, the resuspension of norovirus has also been recognized as one of the transmission modes of gastrointestinal illness. For example, one study investigated the determinant factors of a norovirus outbreak in a concert hall.^[^ ^55^ ^]^ Here, the resuspension of contaminated droplets by air vortices generated from walking across the contaminated zone was speculated to be the primary transmission mode of norovirus.

Resuspension of SARS‐CoV‐2 containing droplets due to human activity has also been investigated. A field report from Wuhan, China, quantified aerosol samples from medical staff areas in hospitals.^[^ ^56^ ^]^ An increased concentration of airborne SARS‐CoV‐2 was observed in staff areas due to resuspension of virus‐laden droplets. It was hypothesized that airflow generated from personnel movements propagated aerosols from patient rooms to staff areas. The highest airborne concentrations, 18 to 42 copies m^–3^ of SARS‐CoV‐2 viral RNA, were detected in the protective apparel changing rooms. This was attributed to the resuspension of settled virus‐laden aerosols by vibrational forces induced by doffing of protective equipment by medical staff who had been in close proximity to infected patients (Figure 3B). Similarly, studies have shown that medical staff uniforms are a notable contributor to bacterial flora causing cross‐contamination.^[^ ^57^ ^]^ Therefore, personal protective equipment can serve as a secondary infection source for healthcare workers. This renders a critical insight into the necessity of effective decontamination strategies for protective apparel and medical uniforms before removal. Alternatively, strategies reducing viral adhesion to PPE could effectively prevent settling of droplets, thereby preventing the possibility of resuspension. In another study conducted in a medical center,^[^ ^58^ ^]^ viral RNA was detected on the ridges of windows and floors beneath patient beds due to the resuspension caused by personnel movements. More importantly, 66.7% of air samples collected from the hallway (∼2.59 copies/L of air) tested positive for viral RNA. Scientists attributed these observations to the resuspension of aerosols by air vortices generated from a door opening as shown in Figure 3D.

Surface roughness is a critical factor that influences the adhesion of a droplet to the substrate. Smooth surfaces increase the contact area at the droplet–surface interface and lead to strong adhesion. Conversely, droplets settled on rougher surfaces experience weak adhesion forces, allowing them to resuspend more effortlessly.^[^ ^59^ ^]^ Surface roughness also determines the initial rolling of droplets during the first phase of resuspension. In a study, the resuspension of droplets was compared between different substrate surfaces such as glass, hardwood, and ceramic. Particles deposited on glass, and ceramic surfaces experienced a longer rolling motion and required high energy to resuspend from the smooth surfaces in contrast to hardwood.

Surface coatings can modulate substrate properties such as surface energy and wettability and therefore determine the fate of resuspension. Floor carpets are usually coated with fluorocarbon and linoleum to reduce the surface energy and the wettability of the fibers to facilitate easy removal of dust or dirt.^[^ ^60^ ^]^ However, these coatings can promote resuspension of settled droplets due to tiny air currents or mechanical forces generated by activities such as vacuum cleaning,^[^ ^61^ ^]^ as shown in Figure 3C.

### 4.2 Effect of environmental factors, humidity, and airflow

As discussed in Section 2, relative humidity is detrimental to virus spread, primarily due to the low survival rate of viruses under high humidity.^[^ ^27^, ^62^ ^]^ However, the presence of water can still enhance virus spread indirectly by disrupting air filtration. For example, Kim et al. showed that the time required to remove particulate matter (PM) from a chamber increased noticeably in wet semi‐HEPA filters compared to dry filters.^[^ ^63^ ^]^ Additionally, dust‐borne transmission has been established as an important route for disseminating avian viruses. Although avian influenza outbreaks have been primarily associated with poultry and bird migrations, outbreaks in Japan, South Korea, and Taiwan have coincided with Asian dust storm (ADS) seasons. Chen et al. quantified airborne virus concentration during ADS and non‐ADS days and found 20‐ to 30‐fold higher virus concentration on ADS days compared to background days.^[^ ^64^ ^]^ This result further implicates dust as an important vector for long‐distance transmission. Moreover, a group of scientists studied the epidemiological factors contributing to the outbreak of *B. anthracis* and concluded that windborne resuspension of anthrax pathogen was one of the supplementary causes of the outbreak.^[^ ^65^ ^]^ In an indoor environment, heating, ventilation, and air conditioning (HVAC) systems are usually considered to improve air quality by diluting, filtering, and removing dust and contaminants. However, recent studies suggest that HVAC systems may enable the infiltration, entrapment, and distribution of respiratory pathogens (Figure 3E).^[^ ^66^ ^]^ In the ongoing COVID‐19 epidemic, samples collected from the air exhaust outlets in a Wuhan hospital tested positive for SARS‐CoV‐2, suggesting that airflows may resuspend virus‐laden droplets and subsequently deposit them on equipment such as vents,^[^ ^67^ ^]^ as depicted in Figure 3F.

Installation of portable air cleaners and equipping furnace and HVAC systems with filters have been suggested as strategies to remove dust and contaminants from indoor air.^[^ ^68^ ^]^ Filters operate on the principles of interception, inertial impaction, and diffusion. Larger particles are intercepted based on size alone, while smaller particles are equally deterred from entry due to the longer path to navigate around fibers. HVAC filters are rated based on their pore sizes and filtering efficiency. However, most commercial filters exhibit a dramatic dip in efficiency against particles in the 0.1–0.3 µm range.^[^ ^69^ ^]^ In commercial airlines and hospitals, high‐efficiency particulate air (HEPA) filters are used to ensure higher air quality. HEPA filters are regulated by US government standards to be able to remove 99.97% of 0.3 µm particles. A NASA study shows that typical HEPA filters are nearly 100% efficient at capturing larger and smaller particles than 0.3 µm.^[^ ^70^ ^]^ However, the capture efficiency declines to 99.97% at the 0.3 µm mark. This is known as the most penetrating particle size (MPPS). The particle sizes of the coronavirus family, which includes SARS‐CoV‐2 and MERS‐Cov, range from 0.06 to 0.2 µm, which is well under the MPPS for commercial HEPA filters. However, viral particles may adhere to aggregates and dust particles to give a final size of 0.3 µm. Therefore, these particles still have a 0.03% probability of escaping HEPA filters. The penetration of viral particles through HEPA filters has been demonstrated by Heimbuch et al. in a laboratory setting.^[^ ^71^ ^]^ Thus, indoor spaces such as airplanes and hospitals are susceptible to virus entry and redistribution even when equipped with HEPA filters, and appropriate decontamination strategies must be adopted to reduce transmission risk.

## 5 MATERIALS TO PREVENT SECONDARY SOURCES OF INFECTION FROM RESPIRATORY DROPLETS

Both primary and resuspended aerosols serve as sources of respiratory infection and necessitate effective approaches to prevent further transmission. A myriad of innovative materials with antiviral properties have been studied recently, driven in part by the COVID‐19 pandemic.^[^ ^72^ ^]^ This section provides a high‐level overview of the material perspective on current applications of antiviral agents in PPE, medical equipment, consumer products, and public settings. A broad array of natural and synthetic materials, including polymer and metals, that can be utilized as surface coatings to deactivate and prevent resuspension of viable virus‐laden droplets is discussed.

### 5.1 Types of virucidal materials

Electrostatically charged materials with virucidal properties have been commonly applied as a surface coating. Both cationic and anionic materials appear to be effective, albeit with distinct mechanisms. Cationic materials usually act via viral capsid destruction, while anionic materials bind to the virus and neutralize their spike proteins. Synthetic polymer‐based antiviral materials often consist of N‐alkylated quaternary amine structures with a positive charge to electrostatically attract viruses and disrupt the protein capsid. This category of virucidal polymers involves derivatives of polyethyleneimine and other existing polymers containing nitrogen, such as polyurethanes^[^ ^73^ ^]^ and polyamides,^[^ ^74^ ^]^ post‐modified with alkyl groups to form the quaternary center, and homo‐^[^ ^75^ ^]^ or co‐polymerized^[^ ^76^ ^]^ diallyl quaternary amines. Natural biopolymer dextran sulfate and other polysaccharide‐based or non‐polysaccharide‐based sulfated polymers such as carrageenan and alginic acid have long been known for their antiviral property.^[^ ^77^ ^]^ The polysulfates bind to the positively charged gp120 envelope protein of HIV to intercept heparan sulfate, the host cell target. The wide application of anionic polysulfates extends to other enveloped and non‐enveloped viruses, including influenza^[^ ^78^ ^]^ and rhinoviruses,^[^ ^79^ ^]^ suggesting that they may bind to viral glycoproteins. In particular, carrageenan^[^ ^80^ ^]^ and alginic acid^[^ ^81^ ^]^ have shown applications as virucidal coatings in other settings, including dip‐coating for fresh food to prevent viral and bacterial entry. Anionic, nonionic, cationic, and zwitterionic surfactants have exhibited antiviral activity. This is attributed to the interaction between the surfactants’ hydrophobic tails and the phospholipid bilayers of the viral envelopes, leading to disintegration and viral deactivation. This mechanism is especially important in enveloped viruses, including influenza and SARS‐CoV‐2. Rhamnolipids, sophorolipids, and similar biosurfactants are of particular interest owing to their biodegradability and low ecotoxicity. Recently, two rhamnolipid products, 222A and 222B, were investigated and demonstrated excellent viral deactivation.^[^ ^82^ ^]^ Although surfactants are primarily used to disinfect surfaces, they may also be embedded in fibers to deactivate viruses that come in contact.

Surfaces of polymeric materials have also been shown to achieve antiviral properties through the use of polyphenols. Polyphenols have previously been shown to display antimicrobial activity against human pathogens through direct action against bacteria and suppression of microbial virulence factors.^[^ ^83^ ^]^ Cellulose non‐woven layers were modified by Catel‐Ferreira et al. through conjugation of catechin polyphenol to impart antiviral properties in wipes and mask filters. Compared to the control, the use of chemically modified wipes decreased the bacteriophage population by 2‐log against a T4D bacteriophage suspension.^[^ ^84^ ^]^ Additionally, virus filtration experiments were performed to test the viability of catechin polyphenol modified filter‐based applications against an aerial suspension of T4D bacteriophage virus. The study results highlighted antiviral properties of polyphenols and potential applications of their presence on polymeric materials.

Nanomaterials have also been explored as virucidal agents, as summarized in Table 2. Given similarities to other enveloped viruses, previously established antiviral mechanisms provide a gateway to the potential application of nanoparticles to prevent pathogen transmission and infection of SARS‐CoV‐2. Virucidal mechanisms of nanomaterials include oxidation damage from reactive oxygen species, competitive binding to host cell receptors, and viral membrane disruption.

**TABLE 2:** Nanomaterial approaches for deactivating respiratory viruses

**Virus**	**Material**	**Mechanism**
Influenza A	Cu	Genomic material is degraded; DNA is compromised by Cu ions, which can bind and cross link between/ within strands^[^ ^126^ ^]^
	Silica/30 nm silver particles	Interaction of free radical generated from AgNPs and Ag+ ions with vial membrane^[^ ^127^ ^]^
	Silver nanoparticles/chitosan	Prevents binding of virus and host^[^ ^128^ ^]^
	Chitin nanofiber sheet‐immobilized silver nanoparticles	Silver can prevent virus–host cell binding^[^ ^129^ ^]^
	TiO_2_‐modified hydroxyapatite	Photocatalytic properties degrading microbe^[^ ^130^ ^]^
	Zinc oxide nanoparticles	Interfere with the influenza virus’ life cycle post viral absorption^[^ ^131^, ^132^ ^]^
Human coronavirus 229E	Cu	Genomic material is degraded; DNA is compromised by Cu ions, which can bind and cross link between/within strands^[^ ^133^ ^]^
	Cu(89%)‐Ni alloy	Genomic material is degraded; DNA is compromised by Cu ions, which can bind and cross link between/within strands^[^ ^133^ ^]^
	Brass (70% Cu, 30% Zn)	Genomic material is degraded; DNA is compromised by Cu ions, which can bind and cross link between/within strands^[^ ^133^ ^]^
SARS‐coronavirus	Ag/Al_2_O_3_	Prevention of viral replication and propagation^[^ ^134^ ^]^
	Cu/Al_2_O_3_	Prevention of viral replication and propagation^[^ ^134^ ^]^
SARS‐CoV‐2	Si_3_N_4_	RNA structural damage leads to viral protein modifications^[^ ^135^ ^]^
	Silver nanoparticles	Infection is impeded either by inhibition of viral attachment and entry, or by weakening viral structural integrity through modifications to the surface proteins^[^ ^136^ ^]^
	Fe_2_O_3_/Fe_3_O_4_	Disrupt viral spike protein's ability of host cell attachment^[^ ^137^ ^]^
	Copper oxide particles	Particle interaction with virions^[^ ^138^ ^]^
	Zinc ions	Inactivation of virus surface proteins^[^ ^132^ ^]^

Metal nanoparticles, including silver, copper, and gold, have previously shown antiviral efficacy over a range of enveloped viruses.^[^ ^85^ ^]^ Metal nanoparticles interfere with viruses’ ability to locate and bind to host cells either by modification or competitive inhibition of specific recognition sites on the capsid. These approaches either utilize the metal's inherent properties or act through grafted pendant functionality. Unmodified gold naturally cleaves disulfide bonds in hemagglutinin, an influenza surface protein, preventing the virus attachment to host cells.^[^ ^86^ ^]^ In particular, silver nanoparticles have been extensively used and reviewed elsewhere and can both inactivate and capture viruses. Similar disulfide interactions occur between silver nanoparticles and dengue virus.^[^ ^87^ ^]^ Although metal nanoparticles do not exhibit specific interaction with viruses, grafting functional groups can be used to impart specificity. For instance, mercaptoethanesulfonate is widely used as a mimic for heparan sulfate and has been employed against the herpes simplex virus,^[^ ^88^ ^]^ coronaviruses, and arteriviruses.^[^ ^89^ ^]^ Although pristine gold cleaves disulfide bonds, gold nanoparticles were reported to attenuate the antiviral activity against influenza. However, grafting of sialic acid to gold nanoparticles impedes the binding of virus hemagglutinin to sialic acid receptors of the host, thereby reinstating the antiviral property.^[^ ^90^ ^]^

Another strategy of viral deactivation is the use of metal oxides such as TiO_2_ and Zn, which generate excess reactive oxygen species (ROS). Multiple studies have demonstrated the virucidal properties of ROS either by direct oxidative damage leading to virus disintegration or various other non‐oxidative pathways.^[^ ^91^ ^]^ SARS‐CoV‐2 is susceptible to reactive oxygen‐mediated photodegradation by TiO_2_ and Zn nanoparticles.^[^ ^92^ ^]^ Park et al. evaluated the virucidal efficacy of surface coatings formulated from photo‐activated fluorinated TiO_2_ against human norovirus and showed effective inhibition of MS2 (positive‐sense single‐stranded RNA material) when exposed to residual ultraviolet A (UVA).^[^ ^93^ ^]^ These findings highlight the potential use of TiO_2_ and Zn nanoparticle surface coatings to sterilize surfaces outdoors in sunlit areas or indoors under fluorescents.

Additionally, pristine metallic nanoparticles have been used against both enveloped and non‐enveloped viruses.^[^ ^94^ ^]^ However, the virucidal property of nanoparticles may be compromised depending on the nature of the coating material. Iron oxide nanoparticles bind and interfere with sulfur‐bearing residues on proteins presented in viral–host cell entry.^[^ ^95^ ^]^ Silver, iron, other metal nanoparticles, or the combination bind in vulnerable disulfide regions of SARS‐CoV‐2 spike protein and thus inactivate the virus.^[^ ^96^ ^]^ A hybrid coating containing metal cations such as copper, iron, and zinc displayed a strong broad‐spectrum antibacterial effect against nosocomial infections. These coatings can be applied in healthcare settings to protect frequently touched surfaces.^[^ ^97^ ^]^

Graphene‐based nanomaterials have also been widely explored for antiviral applications. Graphene oxide (GO) and reduced graphene oxide (rGO) nanosheets contain negatively charged groups which contribute to their antiviral properties. Specifically, these groups bind to virions through electrostatic interactions and subsequently deactivate them through their nanoscale sharp edges.^[^ ^98^ ^]^ Graphene composites with antimicrobial and antiviral metals have been investigated as surface coatings. Jana et al. demonstrated the efficacy of copper–graphene nanocomposites as an antiviral coating by dip‐coating tempered glass in a copper–graphene solution.^[^ ^99^ ^]^ Another study demonstrated that surfaces modified with graphene using plasma‐enhanced chemical vapor deposition (CVD) prevented biofilm formation through the vertical orientation of the graphene nanosheets. The nanosheets protrude from the surface, penetrating bacterial cells and thereby preventing their attachment.^[^ ^100^ ^]^

The use of nanoparticles as virucidal agents would be advantageous in PPE, such as face masks and face shields. Notably, the use of nanoparticles such as copper and gold as additives in 3D printing carries a significant potential to produce more antiviral PPE. 3D printing has been realized as a vital technology in the fight against COVID‐19, during which conventional production chain and distribution systems for PPE faced significant challenges in ramp‐up. To meet supply demands, 3D printing, also known as additive manufacturing, emerged as a means to fill the gap.^[^ ^101^ ^]^ However, a prominent disadvantage of these 3D printed surfaces is that they exhibit high virus attachment and stability for up to 72 h.^[^ ^102^ ^]^ The incorporation of virucidal nanoparticles in the polymer matrix used for 3D printing can help overcome this challenge. The functionality imparted by the nanoparticles will depend on the number of nanoparticles added and printing homogeneity.^[^ ^103^ ^]^ For example, antimicrobial medical devices can be engineered using copper nanocomposite additives in the polymer solution to produce an antimicrobial PLA filament. 3D printing technology can also be used to fabricate facemasks with anti‐viral activity.^[^ ^102^ ^]^ Incorporation of nanoparticles such as TiO_2_ and graphene could potentially impart photothermal functionality, thereby allowing for the effective manufacturing of self‐sterilizing PPE.

Integration of wearable biosensors with PPE is another effective approach to enhance virus mitigation. For example, Kinnamon et al. developed a screen‐printed graphene oxide biosensor to detect environmental exposure to influenza A that could be incorporated with clothing, gloves, and other textiles.^[^ ^104^ ^]^ Further development of such biosensors could allow for early detection of virus exposure and alert healthcare workers to decontaminate the PPE before doffing. Fabrication of PPE with both self‐cleaning and sensing capabilities could prove an effective way to protect against respiratory viruses and merits further study.

### 5.2 Application of virucidal agents onto surfaces

Compared to other virucidal materials, polymeric materials have superior practicality due to their rheological properties, which allow deposition by a simple dip‐coating process. This was recently demonstrated by Haldar et al. using N,N‐dodecyl methyl‐polyethyleneimine (PEIs), and derivatives, which were painted onto a glass surface that deactivated influenza viruses with nearly 100% efficiency.^[^ ^105^ ^]^ Similar coating techniques have also been effective on hard polyethylene^[^ ^106^ ^]^ and soft polyester, polyacrylonitrile, and cellulosic materials. Notably, the application of polyethyleneimine coating onto cellulose fibers has given rise to new designs for low‐cost, high‐efficacy medical masks with anti‐viral properties.^[^ ^107^ ^]^ It is of note that most of the known polymeric coatings with antiviral properties are synthetic cationic polymers. However, there is evidence that the antiviral effect of the coating may be enhanced by complementing the antiviral layer with an anionic antibacterial polymer in a layer‐by‐layer approach.^[^ ^108^ ^]^ Polymeric materials may be directly grafted onto the substrate. Natechin, a simple polyphenol, has also been grafted onto sanitary wipes to confer virucidal capability.^[^ ^109^ ^]^ Similarly, polyethyleneimine was directly polymerized onto silica particles to confer antiviral activity.^[^ ^110^ ^]^ Other virucidal materials such as metals are more challenging to apply on surfaces than polymers. The most straightforward approach is to formulate these materials as a solution or emulsion with polymers that can be used as a surface coating. This approach has been demonstrated with simple antiviral compounds such as chlorine dioxide. Solutions of silver ions and polymer have also been electrospun into polymeric fibers containing silver particles.^[^ ^111^ ^]^ For materials that cannot be stably contained in a solution or emulsion, nanoparticles can be used first to encapsulate the antiviral agent. In a recent paper by Hodek et al., silver, copper, and zinc were encapsulated in a solution of methyl methacrylate, which was then coated onto a substrate and polymerized into a poly(methyl methacrylate) (PMMA) film containing the metals.^[^ ^87^ ^]^ Yet another approach is the covalent modification of the coating polymer to attach the antiviral material. For example, quaternary ammonium compounds can be immobilized onto hyperbranched polymers and applied to glass and plastic surfaces.^[^ ^74^ ^]^ Bioaerosols deposited on the surface may either become immobilized and unable to resuspend if bound to cell‐mimicking structures on the nanoparticles or become inactivated before leaving the surface.

The advancements of self‐sterilizing materials can also be applied as coatings on the protective personal equipment clothing of health care workers. In one study, a photosensitive antiviral layer of a nanofibrous membrane with polyacrylonitrile and poly(vinyl alcohol‐*co*‐ethylene) generated reactive oxygen species in daylight active vitamin K moiety.^[^ ^112^ ^]^ This surface coating on PPE exhibited excellent antiviral activity and deactivated pathogens in deposited droplets, hence preventing secondary infection. Recently, a dual functioning hydrophilic surface coating was developed to capture incoming pathogen‐laden droplets and prevent the resuspension of droplets from the surface.^[^ ^113^ ^]^ The polymer‐based coating constitutes a mixture of polyelectrolyte PAAm‐DDA and non‐ionic surfactant alkyl polyglycosides. The study demonstrated the versatility of the coating, with the ability to be applied to substrates with different material compositions, wettability, roughness, and geometries. Moreover, the incorporation of Cu2+ ions renders an antiviral property to the coating.^[^ ^113^ ^]^ Similarly, coatings and chemical modification of fibers have enhanced the HEPA filter efficiency. HEPA and HVAC filters can be covered with hydrophilic coatings to improve the capture of virus‐laden aerosols and thus prevent the escape and redistribution of pathogens into airstreams.^[^ ^114^ ^]^ In another study, functionalization of polyphenols like tannic acid on filters exhibited superior capture efficiency of influenza virus up to 2723 pfu/mm**^2^** in 10 min compared to the non‐functionalized HEPA filter.^[^ ^115^ ^]^ In general, indoor floor carpets exhibit a high resuspension rate of pathogen‐laden aerosols. Studies have shown that floors covered with polyvinyl chloride significantly reduce the resuspension of *Bacillus atrophaeus* spores from the surface.^[^ ^116^ ^]^ Moreover, addition of fluorosurfactants to the floor finishing materials can enhance the surface wettability by reducing its interfacial tension. These surface coatings are speculated to increase the adhesion force between the settled respiratory droplet and the floor, minimizing resuspension.

Superhydrophobicity has also been explored as a means to prevent adhesion of viral particles onto a surface. Superhydrophobic surfaces, characterized by high water contact angles, replace solid–liquid contact with liquid–air interfaces, thereby lessening contact of the surface with the viral‐laden droplets. In a study by Zhu et al., a coating solution of 2.5 w/w% hydrophobic silica nanoparticle dispersed in ethanol was prepared and applied by dip coating onto different substrates, including glass, plastic, and face masks. In these coated surfaces, SARS‐CoV‐2 attachment was decreased by 99.99995% compared to the bare surfaces.^[^ ^117^ ^]^ Similarly, superhydrophobicity imparted by a transparent coating of alcohol‐based suspension of perfluorinated silica nanoparticles reduced the contact of respiratory droplets on the surface of face shield.^[^ ^118^ ^]^ In combination with self‐sterilizing technologies that can inactivate any residual viruses, superhydrophobic materials demonstrate remarkable efficacy in preventing the possibility of resuspension. For example, Lin. Et al. developed a mask based on graphene nanosheet‐embedded carbon (GNEC) films. GNEC was produced by ultra‐sonic extrusion and then distributed on melt‐blown fibers of thermoplastics, including polypropylene. The surface demonstrated superhydrophobicity and could inactivate any remaining virus due to its photosterilizing ability when exposed to sunlight.^[^ ^119^ ^]^ With an improved understanding of the risk posed by resuspension, careful selection of materials and surface modifications should be considered an instrumental strategy in minimizing secondary infection sources.

## 6 CONCLUSION

Respiratory infections are mainly transmitted via virus‐laden droplets and aerosols. Our review examined the key mechanisms of aerosol generation, ranging from direct mechanisms such as coughing and sneezing to more indirect mechanisms such as medical procedures and toilet plumes. We have also discussed secondary aerosolization, which is influenced by human activities and environmental factors such as temperature and humidity. A wide range of material technologies can be implemented to prevent viral transmission via bioaerosols. We have discussed recent trends and breakthroughs in materials‐based approaches for countering disease transmission through bioaerosols. For example, metal nanoparticles have shown excellent virucidal properties and are particularly effective in surface layer applications, immobilizing bioaerosols and preventing further resuspension. The emerging use of nanotechnology and other virucidal materials in the fight against COVID‐19 and other respiratory diseases dominated by airborne transmission is of notable interest. While current PPE provides some level of protection to users, transmission is yet made possible by contact, for example, touching mucosal surfaces after removing a mask. The resuspension of aerosols from droplets that were previously deposited on surfaces poses a significant risk of disseminating infection. This is especially the case with SARS‐CoV‐2, which demonstrates long survival times on surfaces. Therefore, the use of virucidal coatings and different nanomaterials can be an effective strategy for mitigating transmission. The current strategy of prevention rely on the use of single‐use PPE or sterilization by chemical means (e.g., bleach, H_2_O_2_), which is unsustainable and leads to environmental toxicity. Self‐cleaning materials based on photothermal or photochemical properties provide an alternative to the conventional means of sterilization and significantly reduce chemical waste due to their reusability. Superhydrophobic coatings that prevent adhesion of respiratory droplets on surfaces also prove to be effective strategies in hindering fomite transmission and infection risk by the resuspension of viral‐laden aerosols.

The use of nanoparticles with virucidal properties can be coupled with advancements in additive manufacturing. The addition of virucidal nanocomposites into the polymer matrix used for 3D printing can lead to the production of self‐sterilizing PPE, which will be pivotal in mitigating virus transmission. The integration of electrospun nanofibers in indoor air filters can be an effective approach to improving the filtration efficiency of virus‐laden aerosols. The small diameter and layer thickness of nanofibers reduce the airflow resistance and increase the inertial impaction of the particles across the filter. However, a significant drawback of these fibers is their reduced filtering capacity. Increasing the filter surface area by adopting different shapes over flat filters in HEPA can bypass this challenge. The functionalization of electrospun nanofibers with antiviral materials in HEPA or HVAC filters by dip coating or chemical crosslinking can deactivate the captured virus‐laden droplets. Therefore, installing such filters can effectively improve filtration efficiency and attenuate the transmission risks in indoor spaces such as airplanes and hospitals. Careful consideration of the existing and emerging technologies will allow for effective countermeasures to prevent the resuspension of viral droplets. These technologies can prove instrumental in preventing virus dissemination and hold potential against pandemic outbreaks. Overall, we expect this review to offer a roadmap for preventing respiratory viral infections by providing a greater understanding of bioaerosol‐based transmission mechanisms and suggesting effective countermeasures.

## CONFLICT OF INTEREST

The authors declare no conflict of interest.
